# 3-Amino-1-phenyl-1*H*-benzo[*f*]chromene-2-carbonitrile

**DOI:** 10.1107/S1600536813004376

**Published:** 2013-02-20

**Authors:** Mehmet Akkurt, Alan R. Kennedy, Shaaban Kamel Mohamed, Sabry H. H. Younes, Gary J. Miller

**Affiliations:** a Department of Physics, Faculty of Sciences, Erciyes University, 38039 Kayseri, Turkey; b Department of Pure & Applied Chemistry, University of Strathclyde, 295 Cathedral Street, Glasgow G1 1XL, Scotland; c Chemistry and Environmental Division, Manchester Metropolitan University, Manchester M1 5GD, England; d Chemistry Department, Faculty of Science, Minia University, 61519 El-Minia, Egypt; e Department of Chemistry, Faculty of Science, Sohag University, 82524 Sohag, Egypt; f Analytical Sciences, Manchester Metropolitan University, Manchester M1 5GD, England

## Abstract

In the title compound, C_20_H_14_N_2_O, the phenyl ring is almost normal to the naphthalene ring system with a dihedral angle of 86.72 (9)°. The 4*H*-pyran ring fused with the naphthalene ring system has a boat conformation. In the crystal, mol­ecules are linked into a helical supra­molecular chain along the *b* axis *via* N—H⋯N hydrogen bonds. The chains are consolidated into a three-dimensional architecture by C—H⋯π inter­actions.

## Related literature

For biological and industrial applications of chromene compounds, see, for example: Ellis & Lockhart (2007 ▶); Horton *et al.* (2003 ▶). For puckering parameters, see: Cremer & Pople (1975 ▶). For the graph-set analysis of hydrogen bonding, see: Bernstein *et al.* (1995 ▶).
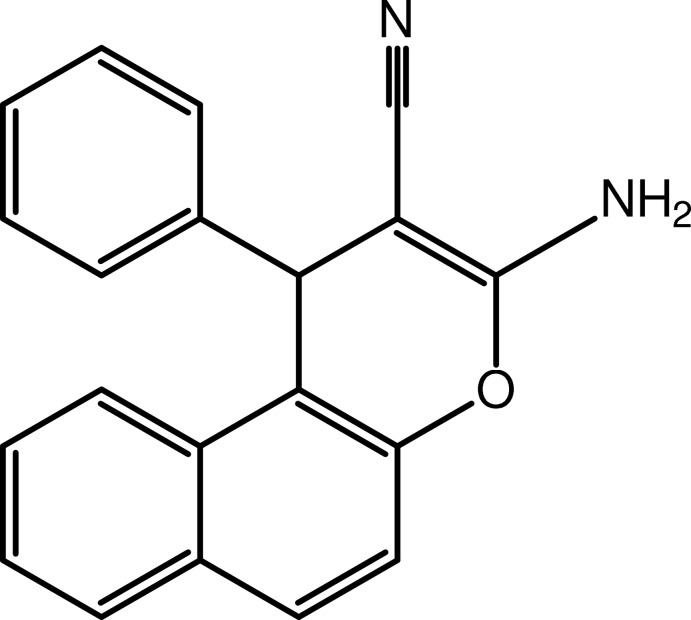


## Experimental

### 

#### Crystal data

C_20_H_14_N_2_O
*M* *_r_* = 298.33
Monoclinic, 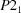

*a* = 9.4059 (8) Å
*b* = 6.5009 (5) Å
*c* = 12.4919 (10) Å
β = 105.914 (9)°
*V* = 734.57 (11) Å^3^
*Z* = 2
Mo *K*α radiation
μ = 0.09 mm^−1^
*T* = 123 K
0.30 × 0.12 × 0.07 mm

#### Data collection

Oxford Diffraction Xcalibur Eos diffractometer
Absorption correction: multi-scan (*CrysAlis PRO*; Oxford Diffraction, 2010 ▶) *T*_min_ = 0.955, *T*_max_ = 1.000
3674 measured reflections
2780 independent reflections
2477 reflections with *I* > 2σ(*I*)
*R*_int_ = 0.019

#### Refinement

*R*[*F* ^2^ > 2σ(*F* ^2^)] = 0.042
*wR*(*F* ^2^) = 0.091
*S* = 1.06
2780 reflections
217 parameters
49 restraints
H atoms treated by a mixture of independent and constrained refinement
Δρ_max_ = 0.22 e Å^−3^
Δρ_min_ = −0.18 e Å^−3^

### 

Data collection: *CrysAlis PRO* (Oxford Diffraction, 2010 ▶); cell refinement: *CrysAlis PRO*; data reduction: *CrysAlis PRO*; program(s) used to solve structure: *SHELXS97* (Sheldrick, 2008 ▶); program(s) used to refine structure: *SHELXL97* (Sheldrick, 2008 ▶); molecular graphics: *ORTEP-3 for Windows* (Farrugia, 2012 ▶); software used to prepare material for publication: *WinGX* (Farrugia, 2012 ▶) and *PLATON* (Spek, 2009 ▶).

## Supplementary Material

Crystal structure: contains datablock(s) global, I. DOI: 10.1107/S1600536813004376/tk5198sup1.cif

Structure factors: contains datablock(s) I. DOI: 10.1107/S1600536813004376/tk5198Isup2.hkl

Supplementary material file. DOI: 10.1107/S1600536813004376/tk5198Isup3.cml

Additional supplementary materials:  crystallographic information; 3D view; checkCIF report

## Figures and Tables

**Table 1:** Hydrogen-bond geometry (Å, °) *Cg*2 and *Cg*3 are the centroids of the C4/C5/C10–C13 and C5–C10 rings, respectively.

*D*—H⋯*A*	*D*—H	H⋯*A*	*D*⋯*A*	*D*—H⋯*A*
N1—H1*N*⋯N2^i^	0.90 (3)	2.16 (2)	2.978 (3)	150 (2)
N1—H2*N*⋯N2^ii^	0.87 (3)	2.33 (3)	3.138 (3)	154 (2)
C7—H7⋯*Cg*3^iii^	0.95	2.84	3.561 (2)	133
C12—H12⋯*Cg*2^iv^	0.95	2.68	3.446 (2)	139

Symmetry codes: (i) 
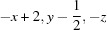
; (ii) 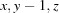; (iii) 
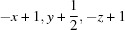
; (iv) 
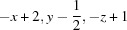
.

**Fig. 1.**
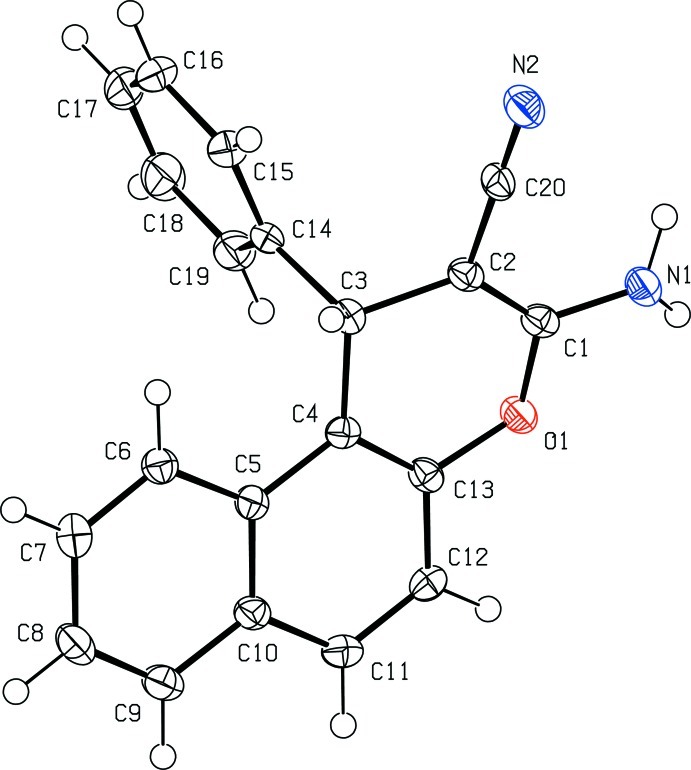
The molecular structure of (I), showing the labelling of the non-H atoms and displacement ellipsoids drawn at the 50% probability level.

**Fig. 2.**
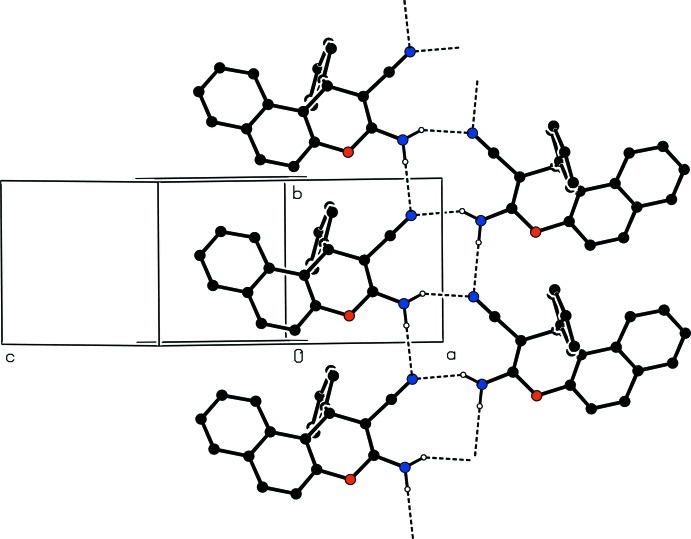
View of the N—H···N hydrogen bonds, having R32(10) ring motifs, forming chains along the b axis. H atoms not involved in hydrogen bonds have been omitted for clarity.

## supplementary crystallographic information

### Comment

Chromenes are components of many natural products (Ellis & Lockhart, 2007) and incorporated in numerous medicinal drugs as significant chromophores. They have shown to display anti-viral, anti-tumoral, anti-anaphylactic, spasmolytic, diuretic and clotting activity (Horton *et al.*, 2003). Furthermore, they can be used as photo-active materials, biodegradable agrochemicals and pigments. As a part of our structural investigations on functionalized chromenes and compounds containing the benzopyran fragment, the single-crystal X-ray diffraction study on the title compound was carried out.

In the title compound (I), Fig. 1, the C14–C19 phenyl ring and the C4–C13 naphthalene ring system is essentially planar with the maximum deviations of -0.004 (2) Å for C16 and 0.015 (2) Å for C4, respectively. They make a dihedral angle of 86.72 (9)° with each other.

The 4*H*-pyran ring (O1/C1–C4/C13) in (I) is puckered with the puckering parameters (Cremer & Pople, 1975) of Q_T_ = 0.211 (2) Å, θ = 96.2 (5)° and φ = 348.9 (6)°. The N1–C1–O1–C13 and N1–C1–C2–C20 torsion angles are -165.54 (17) and -2.6 (3)°, respectively.

In the crystal structure, molecules are linked into a helical supramolecular chain along the *b* axis *via* N—H···N hydrogen bonds (Table 1). Three distinct molecules are linked by three such connections involving two acceptors to generate a *R*^3^_2_(10) ring motif (Fig. 2; Bernstein *et al.*, 1995). Chains are consolidated into a three-dimensional architecture by C—H···π interactions.

### Experimental

Benzylidenepropanedinitrile (1.54 g; 10 mmol) was dissolved in ethanol (50 ml), followed by addition of naphthalen-2-ol (1.44 g; 10 mmol) and a catalytic amount of TEA. The mixture was stirred and refluxed for 2 h at 350 K. The solid product was deposited on cooling at room temperature and collected by filtration. The crude product was washed by cold ethanol, dried under vacuum and recrystallized from ethanol to give high quality crystals (*M*.pt: 563 K) suitable for X-ray analysis in an excellent yield (91%).

### Refinement

All non-hydrogen atoms were refined with anisotropic thermal parameter, however the carbon atoms of the C14–C19 phenyl ring were refined to approximate isotropic behaviour with the "ISOR and DELU" instruction. The H atoms of the NH_2_ group were located by difference synthesis and were refined isotropically. The other H atoms were positioned geometrically, with C—H = 0.95 Å and C—H = 1.00 Å for aromatic and methine H, respectively, and constrained to ride on their parent atoms, with *U*_iso_(H) = 1.2*U*_eq_(C).

### Crystal data

**Table:**

C_20_H_14_N_2_O	*F*(000) = 312
*M**_r_* = 298.33	*D*_x_ = 1.349 Mg m^−^^3^
Monoclinic, *P*2_1_	Mo *K*α radiation, λ = 0.71073 Å
Hall symbol: P 2yb	Cell parameters from 1797 reflections
*a* = 9.4059 (8) Å	θ = 3.1–28.7°
*b* = 6.5009 (5) Å	µ = 0.09 mm^−^^1^
*c* = 12.4919 (10) Å	*T* = 123 K
β = 105.914 (9)°	Rod, colourless
*V* = 734.57 (11) Å^3^	0.30 × 0.12 × 0.07 mm
*Z* = 2	

### Data collection

**Table:**

Oxford Diffraction Xcalibur Eos diffractometer	2780 independent reflections
Radiation source: fine-focus sealed tube	2477 reflections with *I* > 2σ(*I*)
Graphite monochromator	*R*_int_ = 0.019
Detector resolution: 16.0727 pixels mm^-1^	θ_max_ = 28.8°, θ_min_ = 3.2°
ω scans	*h* = −12→10
Absorption correction: multi-scan (*CrysAlis PRO*; Oxford Diffraction, 2010)	*k* = −7→8
*T*_min_ = 0.955, *T*_max_ = 1.000	*l* = −16→16
3674 measured reflections	

### Refinement

**Table:**

Refinement on *F*^2^	Primary atom site location: structure-invariant direct methods
Least-squares matrix: full	Secondary atom site location: difference Fourier map
*R*[*F*^2^ > 2σ(*F*^2^)] = 0.042	Hydrogen site location: inferred from neighbouring sites
*wR*(*F*^2^) = 0.091	H atoms treated by a mixture of independent and constrained refinement
*S* = 1.06	*w* = 1/[σ^2^(*F*_o_^2^) + (0.0342*P*)^2^ + 0.0595*P*] where *P* = (*F*_o_^2^ + 2*F*_c_^2^)/3
2780 reflections	(Δ/σ)_max_ = 0.001
217 parameters	Δρ_max_ = 0.22 e Å^−^^3^
49 restraints	Δρ_min_ = −0.18 e Å^−^^3^

### Special details

**Table:**

Geometry. Bond distances, angles *etc*. have been calculated using the rounded fractional coordinates. All su's are estimated from the variances of the (full) variance-covariance matrix. The cell e.s.d.'s are taken into account in the estimation of distances, angles and torsion angles
Refinement. Refinement on *F*^2^ for ALL reflections except those flagged by the user for potential systematic errors. Weighted *R*-factors *wR* and all goodnesses of fit *S* are based on *F*^2^, conventional *R*-factors *R* are based on *F*, with *F* set to zero for negative *F*^2^. The observed criterion of *F*^2^ > σ(*F*^2^) is used only for calculating -*R*-factor-obs *etc*. and is not relevant to the choice of reflections for refinement. *R*-factors based on *F*^2^ are statistically about twice as large as those based on *F*, and *R*-factors based on ALL data will be even larger.

### Fractional atomic coordinates and isotropic or equivalent isotropic displacement parameters (Å^2^)

**Table:**

	*x*	*y*	*z*	*U*_iso_*/*U*_eq_	
O1	0.91532 (15)	0.1653 (2)	0.28251 (11)	0.0225 (4)	
N1	1.0215 (2)	0.2373 (3)	0.14856 (16)	0.0243 (6)	
N2	0.9541 (2)	0.7742 (3)	0.08334 (15)	0.0294 (6)	
C1	0.9372 (2)	0.3114 (3)	0.21116 (15)	0.0192 (6)	
C2	0.8832 (2)	0.5038 (3)	0.21023 (16)	0.0188 (6)	
C3	0.7813 (2)	0.5686 (3)	0.28021 (16)	0.0173 (6)	
C4	0.7940 (2)	0.4143 (3)	0.37246 (15)	0.0166 (6)	
C5	0.7408 (2)	0.4592 (3)	0.46701 (16)	0.0178 (6)	
C6	0.6672 (2)	0.6459 (4)	0.47667 (16)	0.0207 (6)	
C7	0.6185 (2)	0.6844 (4)	0.56872 (17)	0.0252 (7)	
C8	0.6400 (2)	0.5410 (3)	0.65490 (17)	0.0273 (7)	
C9	0.7100 (2)	0.3578 (4)	0.64841 (17)	0.0246 (7)	
C10	0.7613 (2)	0.3129 (3)	0.55449 (16)	0.0197 (6)	
C11	0.8330 (2)	0.1247 (3)	0.54667 (16)	0.0213 (6)	
C12	0.8812 (2)	0.0815 (3)	0.45537 (16)	0.0197 (6)	
C13	0.8601 (2)	0.2280 (3)	0.37026 (16)	0.0183 (6)	
C14	0.6240 (2)	0.6022 (3)	0.20788 (15)	0.0201 (6)	
C15	0.5843 (2)	0.7921 (4)	0.15674 (16)	0.0275 (7)	
C16	0.4429 (3)	0.8218 (4)	0.08696 (18)	0.0357 (8)	
C17	0.3403 (3)	0.6645 (5)	0.06874 (18)	0.0398 (9)	
C18	0.3793 (3)	0.4760 (5)	0.11953 (19)	0.0368 (8)	
C19	0.5205 (2)	0.4453 (4)	0.18836 (17)	0.0271 (7)	
C20	0.9215 (2)	0.6514 (4)	0.13967 (16)	0.0209 (6)	
H1N	1.024 (3)	0.297 (4)	0.084 (2)	0.038 (7)*	
H2N	1.032 (3)	0.104 (5)	0.146 (2)	0.043 (8)*	
H3	0.81820	0.70340	0.31560	0.0210*	
H6	0.65140	0.74520	0.41880	0.0250*	
H7	0.56950	0.81030	0.57390	0.0300*	
H8	0.60620	0.57030	0.71830	0.0330*	
H9	0.72390	0.26070	0.70720	0.0290*	
H11	0.84780	0.02730	0.60540	0.0260*	
H12	0.92820	−0.04580	0.44970	0.0240*	
H15	0.65400	0.90140	0.16960	0.0330*	
H16	0.41660	0.95100	0.05160	0.0430*	
H17	0.24350	0.68570	0.02150	0.0480*	
H18	0.30910	0.36740	0.10720	0.0440*	
H19	0.54670	0.31510	0.22260	0.0330*	

### Atomic displacement parameters (Å^2^)

**Table:**

	*U*^11^	*U*^22^	*U*^33^	*U*^12^	*U*^13^	*U*^23^
O1	0.0282 (8)	0.0207 (8)	0.0215 (7)	0.0020 (7)	0.0116 (6)	0.0011 (7)
N1	0.0270 (10)	0.0260 (12)	0.0227 (9)	0.0022 (8)	0.0118 (8)	0.0002 (9)
N2	0.0353 (11)	0.0279 (11)	0.0302 (10)	0.0016 (9)	0.0179 (9)	0.0030 (9)
C1	0.0177 (10)	0.0237 (12)	0.0151 (9)	−0.0048 (9)	0.0028 (8)	−0.0008 (9)
C2	0.0183 (10)	0.0216 (11)	0.0169 (9)	−0.0027 (8)	0.0054 (8)	0.0008 (9)
C3	0.0177 (10)	0.0169 (11)	0.0179 (9)	−0.0023 (8)	0.0059 (7)	−0.0017 (9)
C4	0.0142 (10)	0.0180 (11)	0.0161 (9)	−0.0032 (8)	0.0019 (7)	0.0004 (9)
C5	0.0126 (9)	0.0225 (11)	0.0178 (9)	−0.0037 (8)	0.0034 (8)	0.0000 (9)
C6	0.0187 (10)	0.0226 (11)	0.0209 (9)	−0.0015 (9)	0.0057 (8)	0.0018 (10)
C7	0.0224 (11)	0.0260 (13)	0.0292 (11)	0.0004 (9)	0.0105 (9)	−0.0024 (10)
C8	0.0269 (12)	0.0371 (15)	0.0207 (10)	−0.0030 (10)	0.0114 (9)	−0.0025 (10)
C9	0.0242 (11)	0.0305 (13)	0.0192 (10)	−0.0030 (10)	0.0063 (8)	0.0029 (10)
C10	0.0169 (10)	0.0248 (12)	0.0164 (9)	−0.0023 (9)	0.0030 (8)	0.0003 (9)
C11	0.0218 (11)	0.0224 (12)	0.0178 (10)	−0.0025 (9)	0.0020 (8)	0.0026 (10)
C12	0.0174 (10)	0.0180 (11)	0.0217 (10)	−0.0002 (9)	0.0019 (8)	0.0013 (10)
C13	0.0185 (10)	0.0211 (12)	0.0152 (9)	−0.0033 (8)	0.0047 (8)	−0.0034 (9)
C14	0.0212 (10)	0.0269 (12)	0.0131 (9)	0.0041 (9)	0.0061 (8)	0.0011 (9)
C15	0.0292 (12)	0.0301 (13)	0.0238 (10)	0.0070 (10)	0.0085 (9)	0.0055 (11)
C16	0.0358 (13)	0.0479 (16)	0.0227 (11)	0.0204 (12)	0.0069 (10)	0.0096 (12)
C17	0.0227 (12)	0.073 (2)	0.0219 (11)	0.0134 (13)	0.0033 (9)	0.0024 (14)
C18	0.0208 (11)	0.0592 (17)	0.0291 (12)	−0.0029 (12)	0.0046 (9)	−0.0022 (13)
C19	0.0231 (11)	0.0346 (14)	0.0226 (10)	−0.0019 (10)	0.0044 (9)	−0.0003 (11)
C20	0.0223 (10)	0.0235 (11)	0.0181 (9)	0.0019 (9)	0.0077 (8)	−0.0035 (10)

### Geometric parameters (Å, º)

**Table:**

O1—C1	1.356 (2)	C11—C12	1.367 (3)
O1—C13	1.396 (2)	C12—C13	1.400 (3)
N1—C1	1.346 (3)	C14—C19	1.385 (3)
N2—C20	1.160 (3)	C14—C15	1.393 (3)
N1—H1N	0.90 (3)	C15—C16	1.389 (3)
N1—H2N	0.87 (3)	C16—C17	1.381 (4)
C1—C2	1.349 (3)	C17—C18	1.382 (4)
C2—C20	1.415 (3)	C18—C19	1.385 (3)
C2—C3	1.523 (3)	C3—H3	1.0000
C3—C4	1.508 (3)	C6—H6	0.9500
C3—C14	1.523 (3)	C7—H7	0.9500
C4—C13	1.365 (3)	C8—H8	0.9500
C4—C5	1.433 (3)	C9—H9	0.9500
C5—C10	1.421 (3)	C11—H11	0.9500
C5—C6	1.419 (3)	C12—H12	0.9500
C6—C7	1.372 (3)	C15—H15	0.9500
C7—C8	1.396 (3)	C16—H16	0.9500
C8—C9	1.374 (3)	C17—H17	0.9500
C9—C10	1.416 (3)	C18—H18	0.9500
C10—C11	1.413 (3)	C19—H19	0.9500
			
C1—O1—C13	117.88 (15)	C15—C14—C19	119.01 (18)
H1N—N1—H2N	111 (2)	C14—C15—C16	120.1 (2)
C1—N1—H1N	122.0 (17)	C15—C16—C17	120.4 (2)
C1—N1—H2N	117.8 (18)	C16—C17—C18	119.7 (2)
O1—C1—C2	122.03 (17)	C17—C18—C19	120.1 (3)
O1—C1—N1	110.54 (17)	C14—C19—C18	120.8 (2)
N1—C1—C2	127.39 (19)	N2—C20—C2	178.9 (2)
C1—C2—C3	123.11 (17)	C2—C3—H3	107.00
C1—C2—C20	118.34 (19)	C4—C3—H3	107.00
C3—C2—C20	118.54 (18)	C14—C3—H3	107.00
C2—C3—C14	111.16 (16)	C5—C6—H6	120.00
C4—C3—C14	114.16 (16)	C7—C6—H6	120.00
C2—C3—C4	108.92 (16)	C6—C7—H7	120.00
C3—C4—C13	121.06 (17)	C8—C7—H7	120.00
C3—C4—C5	121.46 (17)	C7—C8—H8	120.00
C5—C4—C13	117.46 (17)	C9—C8—H8	120.00
C4—C5—C6	122.21 (18)	C8—C9—H9	120.00
C4—C5—C10	119.53 (17)	C10—C9—H9	120.00
C6—C5—C10	118.26 (18)	C10—C11—H11	120.00
C5—C6—C7	120.7 (2)	C12—C11—H11	120.00
C6—C7—C8	120.9 (2)	C11—C12—H12	121.00
C7—C8—C9	120.24 (19)	C13—C12—H12	121.00
C8—C9—C10	120.4 (2)	C14—C15—H15	120.00
C5—C10—C9	119.60 (19)	C16—C15—H15	120.00
C5—C10—C11	119.53 (17)	C15—C16—H16	120.00
C9—C10—C11	120.88 (19)	C17—C16—H16	120.00
C10—C11—C12	120.61 (18)	C16—C17—H17	120.00
C11—C12—C13	118.90 (18)	C18—C17—H17	120.00
O1—C13—C12	113.06 (16)	C17—C18—H18	120.00
O1—C13—C4	122.98 (17)	C19—C18—H18	120.00
C4—C13—C12	123.96 (18)	C14—C19—H19	120.00
C3—C14—C15	119.59 (17)	C18—C19—H19	120.00
C3—C14—C19	121.37 (18)		
			
C13—O1—C1—N1	165.54 (17)	C5—C4—C13—O1	179.45 (17)
C13—O1—C1—C2	−12.3 (3)	C5—C4—C13—C12	−1.3 (3)
C1—O1—C13—C4	16.6 (3)	C4—C5—C6—C7	−179.59 (19)
C1—O1—C13—C12	−162.72 (17)	C10—C5—C6—C7	0.6 (3)
O1—C1—C2—C3	−6.1 (3)	C4—C5—C10—C9	179.43 (18)
O1—C1—C2—C20	174.79 (17)	C4—C5—C10—C11	−0.4 (3)
N1—C1—C2—C3	176.45 (19)	C6—C5—C10—C9	−0.8 (3)
N1—C1—C2—C20	−2.6 (3)	C6—C5—C10—C11	179.40 (18)
C1—C2—C3—C4	18.5 (3)	C5—C6—C7—C8	−0.1 (3)
C1—C2—C3—C14	−108.1 (2)	C6—C7—C8—C9	−0.3 (3)
C20—C2—C3—C4	−162.42 (17)	C7—C8—C9—C10	0.2 (3)
C20—C2—C3—C14	71.0 (2)	C8—C9—C10—C5	0.4 (3)
C2—C3—C4—C5	164.30 (17)	C8—C9—C10—C11	−179.78 (19)
C2—C3—C4—C13	−14.1 (3)	C5—C10—C11—C12	−0.7 (3)
C14—C3—C4—C5	−70.8 (2)	C9—C10—C11—C12	179.46 (19)
C14—C3—C4—C13	110.8 (2)	C10—C11—C12—C13	0.8 (3)
C2—C3—C14—C15	−85.4 (2)	C11—C12—C13—O1	179.53 (17)
C2—C3—C14—C19	92.2 (2)	C11—C12—C13—C4	0.2 (3)
C4—C3—C14—C15	150.88 (18)	C3—C14—C15—C16	177.37 (19)
C4—C3—C14—C19	−31.5 (3)	C19—C14—C15—C16	−0.3 (3)
C3—C4—C5—C6	3.1 (3)	C3—C14—C19—C18	−177.9 (2)
C3—C4—C5—C10	−177.07 (18)	C15—C14—C19—C18	−0.3 (3)
C13—C4—C5—C6	−178.44 (19)	C14—C15—C16—C17	0.7 (3)
C13—C4—C5—C10	1.4 (3)	C15—C16—C17—C18	−0.6 (4)
C3—C4—C13—O1	−2.1 (3)	C16—C17—C18—C19	0.1 (4)
C3—C4—C13—C12	177.14 (18)	C17—C18—C19—C14	0.4 (4)

### Hydrogen-bond geometry (Å, º)

Cg2 and Cg3 are the centroids of the C4/C5/C10–C13 and C5–C10 rings, respectively.

**Table:**

*D*—H···*A*	*D*—H	H···*A*	*D*···*A*	*D*—H···*A*
N1—H1*N*···N2^i^	0.90 (3)	2.16 (2)	2.978 (3)	150 (2)
N1—H2*N*···N2^ii^	0.87 (3)	2.33 (3)	3.138 (3)	154 (2)
C7—H7···*Cg*3^iii^	0.95	2.84	3.561 (2)	133
C12—H12···*Cg*2^iv^	0.95	2.68	3.446 (2)	139

Symmetry codes: (i) −*x*+2, *y*−1/2, −*z*; (ii) *x*, *y*−1, *z*; (iii) −*x*+1, *y*+1/2, −*z*+1; (iv) −*x*+2, *y*−1/2, −*z*+1.
